# The Monkey Puzzle: A Systematic Review of Studies of Stress, Social Hierarchies, and Heart Disease in Monkeys

**DOI:** 10.1371/journal.pone.0027939

**Published:** 2012-03-21

**Authors:** Mark Petticrew, George Davey Smith

**Affiliations:** 1 Department of Social and Environmental Health Research, London School of Hygiene and Tropical Medicine, London, United Kingdom; 2 Centre for Causal Analyses in Translational Epidemiology (CAiTE), School of Social and Community Medicine, University of Bristol, Bristol, United Kingdom; Texas A&M University, United States of America

## Abstract

**Background:**

It is often suggested that psychosocial factors, such as stress, or one's social position, may play an important role in producing social gradients in human disease. Evidence in favour of this model of health inequalities has relied, in part, on studies of the health effects of the natural social hierarchies found among non-human primates. This study aimed to assess the strength of this evidence.

**Methodology/Principal Findings:**

A systematic review was carried out to identify all studies of psychosocial factors and coronary artery disease (CAD) in non-human primates. We searched databases (MEDLINE, PsycInfo, EMBASE, and Primatelit from inception to November 2010) to identify experimental and observational studies of the impact of social reorganisation, social instability, and disruption of dominance hierarchies on primate CAD outcomes. We also handsearched bibliographies and examined the citations to those studies in public health articles. Fourteen studies were found which presented evidence on CAD and social status and/or psychosocial stress. These suggested that the association between social status and disease may be sex-specific: in female monkeys dominant status may be protective, with subordinate females having a greater extent of atherosclerosis. In male monkeys the reverse may be the case.

**Conclusions/Significance:**

Overall, non-human primate studies present only limited evidence for an association between social status and CAD, Despite this, there is selective citation of individual non-human primate studies in reviews and commentaries relating to human disease aetiology. Such generalisation of data from monkey studies to human societies does not appear warranted.

## Introduction

Socioeconomic gradients in health have been observed in most countries and their existence has been widely accepted by most public health researchers, although the patterns differ for different diseases [1]. There is less agreement about the causes of these patterns, with ongoing debate between proponents of psychosocial and neo-materialist theories. The former group of explanations has been particularly prominent. These suggest that socio-economic position (at an individual level) and income inequality (at a community or country level) influence health primarily through psychosocial mechanisms [2]. A variety of such mechanisms have been discussed – for example, social stress, hostility, lack of control over work and social isolation – with one influential hypothesis being that one's perceived position in the social hierarchy is a particularly important underlying determinant of health and health inequalities [3], [4].

This hypothesis has been supported by international comparisons which suggest that psychosocial factors play an important role in producing social gradients in disease [5]. Conflicting evidence has, however, also been presented, with some evidence that that it may be more important to consider the complex interactions between historical, cultural, economic and other influences on inequalities [6]. The investigation of whether the primary causes of inequalities are psychosocial or materialist has broadened in recent years beyond the study of human populations, to include studies of other primates, such as the old world monkeys, particularly macaques. In such studies researchers based in the US have investigated the impact of social reorganisation, social instability, and the disruption of dominance hierarchies on outcomes such as CAD, and CAD risk factors. For instance, the eminent primatologist Robert Sapolsky's studies of free-living baboon troops in East Africa have found a receptive audience among public health researchers seeking to understand the direct health effects of social status in the absence of material differences in human societies [3], [7], [8], [9], [10], [11], [12], [13].

Generalisation from these non-human, but closely-related primate societies is often seen as appropriate because they are biologically similar to us, develop equivalent diseases, and, like most humans, live in complex, hierarchical societies. Their social organisations are also more amenable to experimental manipulation than human societies; in particular, the effects of deliberate experimental manipulation of social status on coronary arteries can be measured directly in primates in a way that is impossible in humans.

However before one can extrapolate from primate to human societies it is a reasonable to examine the strength of the evidence presented by these studies. There are many possible studies, of different designs, examining differing hypotheses, and not all researchers agree that findings from these studies should be applied to humans. While systematic reviews of animal studies are uncommon, they are important in this as in other fields as a means of assembling and exploring the evidence, exploring inconsistencies and more generally assessing the strength of evidence in support of or against a particular hypothesis. In this case, the review aimed to assess the strength of the evidence describing the association between social hierarchies, social stress and coronary artery disease (CAD) in primates, in the same way that one would review the strength of the epidemiological evidence for an association in studies of humans. We focus on these psychosocial characteristics because these are frequently cited as important mechanisms. A second, related aim of the review was to explore how this evidence has been used in the debate about human inequalities in health.

### From monkey hierarchies to human health

As noted above, the view that one can map such studies in monkeys onto the debate about human inequalities is not without its critics. There is a growing recognition among primatologists that social rank in primates has less to do with physiology than has previously been thought [14]. If this is the case, then theories about human health based on monkey studies may not be well-supported, and it may therefore be timely for research into socioeconomic determinants of health to examine the evidence that psychosocial factors affect health in monkeys, and to consider how this evidence should influence theories about the development of health inequalities in human societies.

We know that in the health literature more generally, “positive” findings are cited much more often than “negative” findings, and that, in general, selective citation and mis-citation can have a distorting effect, overstating the strength of associations between variables and downplaying the importance of studies which do not “fit the argument” [15], [16]. Knowledge of this bias has fostered the use of systematic literature reviews, which attempt to locate, appraise and synthesise all the relevant research evidence on a particular topic, rather than a selected sample of such evidence. However there is no systematic review of the results of the monkey studies to guide public health researchers, although there have been calls for a more systematic approach to the synthesis of animal studies [17].

### What do the primate studies show?

A systematic review of the non-human primate literature provides a basis for assessing the strength of the evidence that status in monkeys predicts ill health, as well as providing an indirect test of the psychosocial hypothesis in humans. An appropriate starting point for such a review is to examine the results of the primate studies of the effect of social stress on actual CAD outcomes. The specific null hypotheses tested here are (i) that there is no association between social hierarchy and CAD outcomes, and (ii) that increased stress in these animals is not associated with increased risk of CAD. These specific hypotheses were chosen because the psychosocial model has drawn upon studies of these mechanisms and outcomes in monkeys to propose possible explanations for health inequalities in humans; although other indirect outcomes are of interest (e.g., health behaviours) it is the direct, negative health effects of hierarchies on disease outcomes that have been the subject of most debate, rather than the indirect effects (e.g., effects on health behaviours).

## Methods

### Search strategy

We undertook an extensive electronic search and hand search of bibliographies in order to identify studies of the impact of social status or social stress on CAD outcomes in monkeys. We searched MEDLINE (1950–Nov 2010), PsycInfo, EMBASE, and Primatelit from inception (i.e., earliest possible start date) up to the end of November 2010, using free text search terms primate* or monkey*, or macaque*, *plus* heart, athero*, disease, artery, coronary, CHD, or CAD, *plus* social stress, stress*, or status. While we accepted that this simple search strategy would produce a large number of irrelevant hits we felt that this would be manageable, and that a thorough search of bibliographies would identify any additional studies missed by the electronic searches. We therefore conducted an extensive search of the bibliographies of all primary studies, previous review papers, and book chapters.

### Study inclusion criteria

We included studies of social reorganisation, social instability, and disruption of dominance hierarchies. Only studies reporting CAD outcomes (such as extent of arteriosclerosis, size of intimal area, and plaque or lesion size) were included. Studies which reported only risk factors without disease outcomes were excluded. Studies reported in any language were included. Studies of social status/rank, social reorganisation, social instability or social stress with CAD outcomes, as reported by study authors (e.g., extent of atherosclerosis; plaque size; size of intimal area; or other measure of presence or extent of disease) in monkeys were included. Studies with no CAD outcomes (e.g., studies which included only changes in stress hormone or glucocorticoid levels, heart rate reactivity, blood pressure, or lipid levels as outcomes) were excluded. These inclusion criteria were applied by two reviewers working independently.

The intervention being investigated in this review is therefore social reorganisation, social instability, or disruption of dominance hierarchies. The comparison group is either animals whose position in the social hierarchy was not disrupted, or who were at a different position in the hierarchy (e.g. dominant vs subordinate animals are compared). Both experimental and observational study designs were eligible for inclusion.

### Study selection

One reviewer screened the titles and abstracts for possible inclusion to produce a list of 148 possibly relevant studies. The final decision on inclusion was made by two reviewers.

### Quality assessment

Methodological information relating to study quality was tabulated and checked by two reviewers. We used the widely-used Quality Assessment Tool for quantitative studies developed by the Effective Public Health Practice Project at McMaster University (see: http://www.ephpp.ca/Tools.html).

### Data collection process

Data were extracted by one reviewer and checked by a second. These were summarised in the overall summary table (Table 1), with the data on the methodological assessment in Table 2; the full data appear in Table S1. We included numbers and sex of animals in the study, study design, CAD outcome data where reported, and the statistical significance of any test where this was reported. Effect size data were extracted directly from each paper, or were recalculated where possible.

**Table 1:** Studies of the effects of social stress and/or social status on the development of CAD in non-human primates (full results table available: Table T1).

Study number*, author, year	Sample	Study design & intervention	Results relating to social status or CAD
1. Adams et al. (1985) [26]	52 female cynomolgus monkeys,	Controlled trial, ovariectomy (n = 25) vs intact ovaries (n = 27), atherogenic diet.	*Social status*: Ovariectomised dominant females had more CAA than intact females. No association between dominance/subordination and lesions.
2. Clarkson et al. (1990) [25]	83 Female cynomolgus monkeys,	Trial: 2 groups randomised to oral contraceptives; 1 control group. Atherogenic diet.	*Social status*: Pre-experimental social status predicted atherosclerosis on necropsy (p<0.03, no other data)
3. Hamm (1983) [20], [50]	16 male and 16 female cynomolgus monkeys	Randomly allocated to social groups, then housed in stable single-sex groups. Atherogenic diet.	*Social status*: Coronary artery stenosis greater in males and submissive animals.
4. Kaplan et al. (1982) [27]	30 Male cynomolgus monkeys	Controlled trial: monkeys assigned to unstable or stable social groups (15 monkeys in each group)	*Social status*: No effect of dominance or instability. Dominant monkeys in unstable group had greater atherosclerosis than those in stable group; atherosclerosis greater in unstable dominants vs unstable subordinates.
5. Kaplan et al. (1983) [29], [30]	30 Male cynomolgus monkeys	Controlled trial: 1. stressed (periodically re-organised) (n = 15); unstressed (n = 15). Low fat diet.	*Stress:* Stressed animals had greater CAA than controls.
6. Kaplan et al, (1984) [21]	23 female and 15 male cynomolgus monkeys	Controlled trial: 2 male, stable groups, 2 female groups, one stable, one regularly disrupted. Atherogenic diet.	*Social status:* Greater CAA in males vs dominant females; subordinates more likely to have CAA. No difference between males and subordinate females, or between stable and unstable groups.
7. Kaplan et al. (1987) [18]	30 male cynomolgus monkeys	Controlled trial: propranolol (n = 15) vs untreated (n = 15).Randomised into 5 member groups re-organised monthly. Atherogenic diet.	*Social status*: Untreated dominant monkeys had more CAA. Significant drug treatment × dominance interaction (F_1,20_ = 5.48, p = 0.028).
8. Kaplan et al. (1993) [31]	83 adult male 100 cynomolgus monkeys	Controlled trial. Baseline period with stable groups; atherogenic diet, then stressor introduced (group reorganisation)	*Social stress:* Stressed monkeys had larger lesions.
9. Kaplan & Manuck (2001,2002) [22], [51], [52]	175 female cynomolgus monkeys	Controlled trial. Half the groups randomly received oral contraceptives. Monkeys oophorectomised, then 36 month post-menopausal period, followed by necropsy. Atherogenic diet.	*Social status*: Untreated dominant monkeys had less CAD than untreated subordinates (p = 0.001).
10. Shively et al.(1989, 1990) [23], [53]	77 female cynomolgus monkeys	2×2 controlled trial: Single cage vs social housing, and oral contraceptive vs no OC. Atherogenic diet.	*Social status*: Untreated controls: Single monkeys had more CAA than socially dominant females but not socially subordinate females.
11. Shively & Clarkson (1994) [28]	48 adult female cynomolgus monkeys	Monkeys randomly allocated to groups until social status stabilised. Then groups reorganised to produce 4 groups: 1. Initially dominant, remained dominant after regrouping; 2. Initially dominant, then subordinate; 3. Initially subordinate, then dominant ; 4. Initially subordinate, then subordinate. Atherogenic diet.	*Social status*: Among initially subordinate females, those becoming dominant had more extensive CAA and among initially dominant females, those who became subordinate had more extensive CAA
12. Williams et al. (1991) [32]	33 male cynomolgus monkeys	Experimental study, factorial design (social disruption × high/low cholesterol diet)	Data only presented for low cholesterol group; no effect of disruption.
13. Williams et al. (1994) [24]	25 female cynomolgus monkeys	Randomly allocated to 4-member stable groups. Atherogenic diet	*Social status*: No significant difference in CAA between dominant and subordinate monkeys.
14. Williams et al. (2003) [33]	71 male cynomolgus monkeys	Factorial design: exercise vs social instability. High-fat diet to model North American diet.	No effect of social reorganisation on CAA.

*Key:* CAA: coronary artery atherosclerosis; CAD: coronary artery disease; OC: oral contraceptive.

**Table 2:** Methodological assessment of the included studies.

Study number, author (year)	Allocation bias (Design, and method of randomization if used)	Confounders with respect to stress/status/CAD relationship reported/analysed/adjusted for	Blinding	Data collection methods (stress/social status, and CAD) valid and reliable	Withdrawals/dropouts	Analysis *(i.power or sample size calculation; ii.significant difference; iii.appropriate statistical methods)*	Intervention integrity *(No evidence of contamination or co-intervention)*
1. Adams et al. (1985) [26]	Observational/aetiological study	No	Blinded outcome assessment	Yes	8/52 (15%) died of causes unrelated to the study	i. Noii. Yesiii. Yes	N/A
2. Clarkson et al. (1990) [25]	Observational/aetiological study within an RCT	Yes	Not reported	Yes	10/83 (12%) died of causes unrelated to the study	i. Noii. Not reported for social status analysisiii. Yes	N/A
3. Hamm (1983) [20] [50]	Observational/aetiological study	No	Not reported	Yes	None	i. Noii. Yesiii. Yes	N/A
4. Kaplan et al. (1982) [27]	Controlled trial (stable vs unstable)	No	Not reported	Yes	2/30 (7%) died	i. Noii. Yesiii. Yes	Yes
5. Kaplan et al. (1983) [29], [30]	Controlled trial	Yes - potential confounders analysed and shown non-significant	Yes	Yes	None	i. Noii. Yesiii. Yes	Yes
6. Kaplan et al, (1984) [21]	Controlled trial (stable vs unstable)	No	Not reported	Yes	4/42 (10%) died of causes unrelated to the study	i. Noii. Not all data presentediii. Yes	Yes
7. Kaplan et al. (1987) [18]	Observational study within controlled trial	No	Not reported	Yes	6/30 (20%) lost to the study, equal numbers from each arm of trial	i. Noii. Yesiii. Yes	N/A
8. Kaplan et al. (1993) [31]	Controlled trial	Yes (serum lipids; blood pressure; body size)	Not reported	Yes	17/100 (17%) lost to the study for reasons unrelated to the study	i. Noii. Yesiii. Yes	Yes
9. Kaplan & Manuck (2001,2002) [22], [51], [52]	Observational study within trial	Plasma lipids	Yes	Yes	36/213 (17%) lost to study for reasons of illness or death	i. Noii. Yesiii. Yes	N/A
10.Shively et al. (1989, 1990) [23], [53]	Observational study within a trial	Total plasma cholesterol/high density lipoprotein	Not reported	Yes	4/77 (5%)died for reasons of illness or death	i. Noii. Yesiii. Yes	N/A
11. Shively & Clarkson (1994) [28]	Observational study within a trial	Yes, incl. Plasma cholesterol, insulin, HDL cholesterol, adiposity, thigh circumference	Not reported	Yes	6/48 (13%) died for reasons unrelated to study.	i. Noii. Yesiii. (See text)	N/A
12. Williams et al. (1991) [32]	Observational study	No	Not reported	Yes	1/33 (3%) died during catheterization	i. Noii. Yesiii. Yes	N/A
13. Williams et al. (1994) [24]	Observational study within trial	TPC, HDL concentrations, SBP and DBP analysed	Not reported	Yes	6/48 (13%) died for reasons unrelated to the study	i. Noii. Yesiii. Yes	N/A
14. Williams et al. (2003) [33]	Factorial design	Heart rate, blood pressure, body weight	Not reported	Yes	20% of 95 animals died pre-study	i. Noii. Yesiii. Yes	Yes

“Selection bias” item not included as it is not possible to determine to what extent the included animals represent a “target population”. Similarly “agreement to participate” - the other element of this item - is not relevant. “Intervention integrity” item from the EPHPP tool is not included, as not all studies employed an intervention.

### Summary measures, and synthesis of results

The main summary measures used in the studies were extent of coronary artery atherosclerosis in mm^2^; % stenosis (for example. “extent of atherosclerosis in arterial section, measured as the area in mm^2^ between the internal; elastic lamina and the lumen” [18]); and % of animals with serious CAD The findings were summarised narratively, given the heterogeneity in study designs and the observational nature of the data.

### Citation search

We hand-searched the full-text and bibliographies of articles and book chapters on health inequalities, and carried out citation searches in Web of Science to identify papers which had cited any of the studies we identified (Table 3). We also checked the references of a 2009 review which summarised the findings of studies conducted by the main research laboratory working in this field [19]. Finally, to inform the discussion section of this paper, a citation search was run on each included study and the titles and abstracts of the citing papers were reviewed.

**Table 3:** Citations of primate studies in public health articles up to 2009.

Study	Number of citations overall	Number (%) on human social epidemiology
Adams 1985 [26]	105	15 (14.3)
Clarkson 1990 [25]	119	4 (3.4)
Hamm 1983 [20]	88	22 (25)
Kaplan 1982 [27]	185	92 (49.7)
Kaplan 1983 [29]	131	62 (47.3)
Kaplan 1984 [21]	90	30 (33)
Kaplan 1987 [18]]	188	48 (25.5)
Kaplan 1993 [31]	23	3 (13)
Kaplan 2002 [51]	42	5 (11.9)
Shively 1989 [23]	34	13 (38.2)
Shively & Clarkson 1994 [28]	60	38 (63.3)
Williams et al. 1991 [32]	46	27 (58.7)
Williams et al. 1994 [24]	44	7 (15.9)
Williams et al. 2003 [33]	8	0 (0)

## Results

### Studies of status, social stress and CAD

Our searches produced 3020 hits (See PRISMA flowchart; Figure S1], from which we identified 14 studies (Table 1; methodological assessment appears in Table 2) which met the inclusion criteria. Social status generally emerges as a result of encounters among individual animals, in some cases following a period of deliberate disruption of the social groups by experimenters. The findings are summarised in these two broad categories below. Ten studies presented information on the relationship between social status (dominance, subordinance) and CAD outcomes. Four studies reported on the effects of social disruption or other social stress. Two studies reported data on both social status, and stress.

### Studies of social status (Table 1)

The ten studies of social status and atherosclerosis reported variously that dominant status is protective of CAD (Study Nos. 3, 6, 9,11) [20], [21], [22], [23], that it is a risk factor for CAD (Study No. 7) [18], and that there is no association (Study No. 13) [24]. One other study of female monkeys presented too few data to assess the relationship (Study No.2) [25]. There may be an interaction with sex, such that subordinate females have a greater extent of atherosclerosis than dominant females (Study Nos. 1, 3, 6, 9,11) [20], [21], [22], [23], [26], while the reverse association has been reported for males, (Study Nos. 4,7) [18], [27] although not in all studies (Study No. 3) [20]. One study (No. 13) [24] of female monkeys reported no difference in the extent of atherosclerosis between subordinate and dominant animals, and another study found that change in social status, in both dominant and subordinate female animals, predicted the extent of atherosclerosis (Study No. 12) [28]. One study reported that there was no main effect for dominance in male monkeys, though dominant monkeys in unstable social conditions developed more severe atherosclerosis (Study No. 4) [27].

### Studies of psychosocial stress

Six studies *(Studies numbered 4–6, 8, 12, and 15 in* *Table 1* *)* examined the effects on CAD of psychosocial stress (generally in the form of experimental disruption to social groups). (Two studies reported data on both social status, and social stressors: Study Nos. 4 [27] and 6 [21]) In one study, male monkeys were separated into unstable (periodically disrupted) and stable groups, and the extent of atherosclerosis was measured at follow-up (Study No. 4) [27] No main effect was found for social stress, although an interaction with status was observed, such that dominant animals had more severe atherosclerosis in conditions of social disruption (this study was also described in the previous section). In a further study in male monkeys (Study No. 5) [29], [30] the same researchers periodically reorganised the animals' social groups and compared these animals to those in a control group; the extent of CAD was found to be greater in the reorganised (stressed) group. This finding was not confirmed in a subsequent study in male and female monkeys (Study No. 6), which found no difference between stable and unstable groups [21]. One study (Study No. 8) [31] found that stressed male monkeys on a high fat diet had more atherosclerosis than stressed monkeys consuming a low fat diet, or unstressed monkeys. Two other studies (Studies 12 [32] and 14 [33]) in adult male cynomolgus monkeys found no associated between stress due to social reorganisation and CAD. One further small study (not tabulated) has reported that social stress caused CAD, but few data were presented [34].

## Discussion

In summary, the studies of social status in primates do not appear to provide strong evidence that social status is an important predictor of CAD in monkeys. Rather, they seem to suggest that the relationships between social status and CAD may be sex-specific, and in particular that subordinate status is a more fallible predictor of atherosclerosis than is sometimes assumed. Indeed, in male monkeys it appears that dominant, not subordinate, status may sometimes be pathological. With respect to the studies which have examined the effects of social stress on CAD outcomes, the studies are suggestive, but there are probably too few data at present to come to any firm conclusions.

One study here is of particular interest, as it is widely cited as providing supporting evidence for a pathological relationship between low social rank and poor health [28]. In this study monkeys were randomly allocated to 4-member groups for 8 weeks until the social rankings stabilised. Dominant monkeys were then housed together, and the subordinates housed together, forming 4 groups in total: females that were initially dominant, and remained dominant after regrouping (n = 11), monkeys that were initially dominant, but became subordinate after regrouping (n = 11); those that were initially subordinate, but became dominant (n = 8) and those that were initially subordinate, and remained so (n = 12). One finding from this study is often cited in support of the psychosocial interpretation of health inequalities, to the effect that among dominants that became subordinate the extent of coronary artery atherosclerosis increased by 500% - which is used as evidence of the harmful effects of low social status (See Table S2). This figure is however based on a comparison between the extent of atherosclerosis among monkeys who stayed at the same rank (either dominant or subordinate) (mean extent of CAA approximately  = 0.035 mm^2^), and those who became subordinate (mean =  0.19 mm^2^) and these data from only 42 monkeys are adjusted for skewness, heterogeneity of variance, and two potential CAD risk factors (thigh circumference and ratio of TPC-HDL). The unadjusted data (as estimated from the original scatterplot shown in Figure S2) seem to show no meaningful association between plaque size, and rank. Moreover, monkeys who were previously subordinate, but became dominant, also experienced an increase in CAA, by 44% greater than the subordinates that stayed subordinate. If any message is to be drawn from this small study, then the message is that change in status in either direction, and not status itself, is pathogenic. The researchers who conducted the study themselves point this out: *“All animals with altered social positions (dominants that became subordinate, and subordinates that became dominant) had worsened coronary artery atherosclerosis”* (page 725) The straightforward “500% increase” claim on its own is therefore misleading; if anything, subordinate monkeys are better off staying subordinate.

Finally, though we did not formally assess publication bias, the risk of this and other biases in this small sample of small studies should also be borne in mind, including outcome reporting bias.

### Use of the primate evidence in public health

Although as we have seen the evidence appears equivocal, these studies are frequently cited in epidemiological and public health literature in support of the “psychosocial hypothesis”, a conceptual model describing the relationships between social stress, human hierarchies, and human health outcomes [35]. Robert Sapolsky's baboons (not included in this review, as objective CAD measures have not been collected on this group) are particularly prominent in these discussions:

*“The Whitehall and Serengeti studies are in a sense starting from opposite ends of a possible bridge. While the baboons show hierarchically associated variations in physiological responses to stress that are consistent with health effects, the civil servants show hierarchical variations in health outcomes that must emerge from some physiological pathway.”* [36]

The experimental studies in macaques are also used to suggest that the low status is harmful in monkeys and by extension, in humans. As illustration, Table 3 shows the frequency of citation of most of the primate studies whose results are described above. A sizeable percentage of citations derive from articles in epidemiology and public health journals, but more interesting is the popularity of the Shively and Clarkson (1994) study described above – most of its citations are by researchers outside of the field of primatology, and in these papers it is often cited in the context of discussion of the direct health effects of human social status [37], [38]. Despite its equivocal findings, this study, more than any other primate study, is used to support the view that low rank in human societies is directly harmful to health. The disconnect between the reality of available non-human primate data and the interpretation given to it by commentators from various disciplines has been commented on in a different context [39].

A common form of citation is to refer to both the Shively and Clarkson study of female monkeys (which studied coronary atheroma), and Sapolsky's study of male monkeys (which examined blood lipid levels) (See Table S2 for examples). This suggests that the finding relating to CAD is robust and also relates to males, but when studies of CAD in male monkeys are examined the results are, if anything, in an opposite direction to those (possibly) found by Shively for female monkeys. A further popular claim is that a dramatic five-fold increase in atherosclerosis was generated by downward mobility in the social hierarchy, again referring to the Shively and Clarkson study, though the unadjusted data show little effect.

### The limits to generalisation

The evidence from these studies does not provide strong support for a psychosocial explanation of health inequalities. The data derive from studies, which are, almost by necessity, small, ranging from 23 to 193 animals. By contrast, the number of statistical tests carried out in these studies is often large, and power calculations to justify the sample sizes are absent. The need for small sample sizes is understandable in primate research, as is the need to include animal participants in more than one study, but similar biases apply to animal studies as apply to other epidemiological studies, including lack of intention to treat analyses, and lack of blinding of outcome assessment in most studies. Drop-outs (e.g. in this case due to animals dying) are however generally low, at least compared to community-based studies in humans. Other unknown observer biases may also be operating; for example, one meta-analysis of field studies of baboon behaviour has reported that observers recording behaviour of troops of baboons had a tendency to favour watching larger groups, which tend to travel less far, and also differ in other behaviours [40].

Primatologists themselves have warned repeatedly about over-generalising from primate data to human societies [12], [41], [42]. Indeed the data may not even be generalisable between similar species of monkey, as comparative research and field studies suggest that there are striking differences in group composition, social spacing, dominance and aggression between species [41]. The social and hierarchical behaviour of *Macaca fascicularis*, the species used in many of these studies, may not therefore even be representative of all of its own genus, which raises doubt about extrapolation to higher primates.

Robert Sapolsky has raised another problem for researchers seeking to generalise to humans. He describes *“the circuitous and often tragic routes by which primates come to find themselves in laboratories…It is not generally known that there is an extremely high mortality rate among primates during transit…survivors may well be those which have passed successfully through what evolutionists refer to a “selective bottleneck” – and either may be physically and psychiatrically robust, or permanently weakened by their vicissitudes…they could well be “supermonkeys”* [43]. Even if this is not the case, he concludes that the primates available for study represent a far-from-random sample.

This in itself represents a limitation of our review, in that it examines evidence from a selected group of animals. Perhaps more importantly in some cases the investigation of social status is not a primary focus of the study (see Table 1, column 1), and in all cases the numbers are small and so they may have had limited power to detect real differences in outcomes between subordinate and dominant (or stressed and unstressed) animals. This heterogeneity in study design and study purpose makes drawing firm conclusions about CAD and social position in primates difficult.

Simplistic interpretations of human hierarchies have also been criticised by Rohde (2001), who agrees that human hierarchies are common, with governments, religions, workplaces, and schools often arranged hierarchically [44]. The term “hierarchy” is of Greek origin, originally referred to ranks of ecclesiastical rulers, and was later used to describe the pecking order of angels. Hierarchies often appear rapidly and spontaneously, and phrases like “pecking order” itself are well–recognised. However interpretation of human hierarchies is often difficult, and Rohde suggests four reasons for this. Firstly, higher-ranking people often make paradoxically submissive gestures, (such as allowing others to pass through a doorway first, and there is the obligation of the strong to care for the weak in some religions and in other forms of social obligation, and for the well to care for the sick). Secondly, human hierarchical aspirations can be expressed purely cognitively, with no obligation to action, because humans, with symbolic thought, can model likely outcomes of confrontations. Thirdly, there is a thick cultural veneer (including manners) which overlays hierarchical relations in humans. Finally, humans may have many alternative hierarchies, with perfectly satisfactory alternative hierarchical positions [43].

### Primate social rank and the stress response

This overview has concentrated on CAD outcomes, but studies have also examined associations between dominance hierarchies and hormonal responses. These markers of stress response also appear to suggest psychoneuroendocrine pathways underlying the development of human inequalities in cardiovascular disease. For example it has been suggested that there is an established relationship between high basal cortisol levels and subordinate status, and that this is consistent across primate species [45]. However a comparative analysis of this issue reached the opposite conclusion. This study involved a systematic review of data on subordinate/dominant cortisol ratios across ten different primate species, and found cortisol ratios associated with dominant/subordinate status to be highly heterogeneous. The authors concluded that there is no consistent relationship between social rank and stress response in primates and argue that there is no generalisable relationship between social status and any aspect of stress physiology across monkey species [46] (Table 4). A recent large study has also found that the highest rank wild male baboons had an unfavourable profile of stress-related hormonal measure, in contradiction to the anticipated favourable effects of hierarchy on such indicators [47].

**Table 4:** Variation in subordinate/dominant cortisol ratios, by primate species [45].

Species	Relative cortisol ratio (%)*
Common marmoset – female	45
Cotton top tamarind– female	80
Cotton top tamarind- male	82
Squirrel monkey – female	98
Rhesus monkey – male	99
Talapoin monkey – female	105
Cynomolgus monkey – female	127
Squirrel monkey – male	145
Olive baboon – male	147
Talapoin monkey – male	154

* The percentage in column 2 represents a comparison between the basal cortisol level in subordinate and dominant animals - e.g., for marmosets, subordinate animals have 45% of the cortisol level of dominant animals.

Finally, this review provides an example of the difficulty in extrapolating from very few or single studies, particularly when those few studies provide conflicting evidence. There are many examples in the literature of where single studies taken out of context may be misleading, which has fostered an awareness of the risks of relying on single studies, and of the need for comprehensive systematic reviews of the evidence. However such reviews are still relatively uncommon outside of the health and social sciences, though the case has been made previously for the need for more systematic reviews of animal research in particular, both to summarise existing literature and to help direct future research [48], [49].

### Conclusions

Two conclusions can be drawn from this review of the monkey evidence on social rank and CAD. The first is that non-human primate studies present limited evidence for an association between rank and CAD in monkeys; the effects of stress, and social status appear to be more inconsistent than is often assumed, and the relationships may be sex-specific. The data presented to support these associations themselves are also limited, deriving from small studies in highly–selected populations. We took the view that the strongest evidence is likely to come from studies which analyse the impact of these psychosocial factors on CAD outcomes, rather than intermediate outcomes. However it may also be informative in future to conduct a systematic review of studies which address factors related to CAD even if they do not directly measure it.

Secondly, generalisation of these data to human societies may not be warranted, and is against the advice of the primatologists conducting such studies. Given the pre-eminence of Robert Sapolsky's Serengeti baboons in the public health literature, it is probably appropriate to conclude with Sapolsky's own views on rank in monkeys, and its application to humans:

*“It seems virtually meaningless to think about the physiological correlates of rank outside the context of a number of other modifiers… This dovetails nicely with the de-emphasis of rank in other niches of primatology …It leads to a final, somewhat obvious point – if we are endlessly struck with the complexity of these issues as they apply to non-human primates, the complexity expands exponentially when considering humans”* [12].

## Supporting Information

Figure S1 **PRISMA Flowchart.**
(TIF)

Figure S2 **Atherosclerosis in female monkeys after change in social status.** Key: A: Dominant animals who stayed dominant ; B: dominants who became subordinate ; C: Subordinates who became dominant; D: Subordinates who stayed subordinate.
(TIF)

Table S1 **Studies of the effects of social stressors and/or social status on the development of CAD in non-human primates.**
(DOC)

Table S2 **Examples of quotations from papers citing primate studies.**
(DOC)

Checklist SI **PRISMA 2009 Checklist.**
(DOC)
