# Supplementary figures and images for: The Monkey Puzzle: A Systematic Review of Studies of Stress, Social Hierarchies, and Heart Disease in Monkeys
Source: PLoS One. 2012 Mar 21;7(3):e27939. doi: 10.1371/journal.pone.0027939 (PMC3309950; doi:10.1371/journal.pone.0027939)

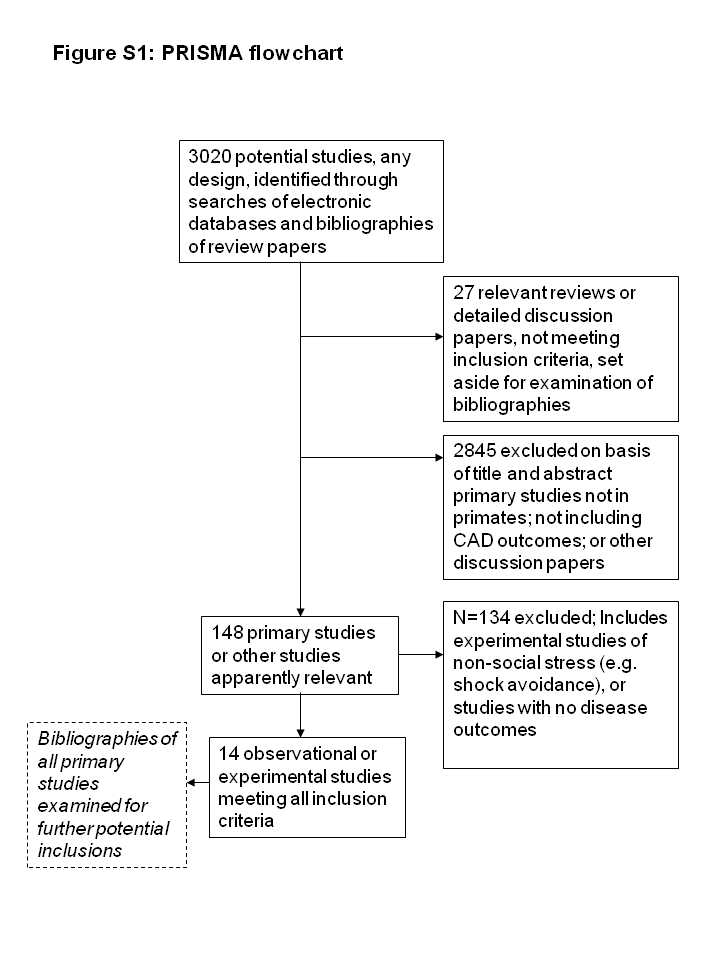

Supplement: Figure S1 — PRISMA Flowchart. (TIF) [file pone.0027939.s001.tif]

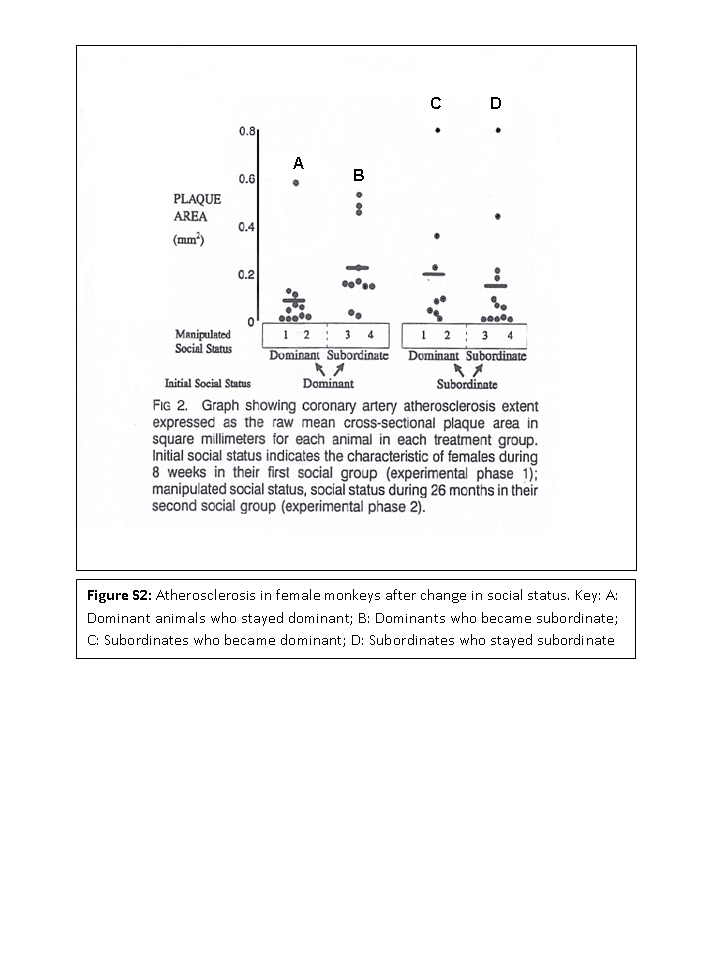

Supplement: Figure S2 — Atherosclerosis in female monkeys after change in social status. Key: A: Dominant animals who stayed dominant ; B: dominants who became subordinate ; C: Subordinates who became dominant; D: Subordinates who stayed subordinate. (TIF) [file pone.0027939.s002.tif]
