# Supplementary material for: The Monkey Puzzle: A Systematic Review of Studies of Stress, Social Hierarchies, and Heart Disease in Monkeys
Source: PLoS One. 2012 Mar 21;7(3):e27939. doi: 10.1371/journal.pone.0027939 (PMC3309950; doi:10.1371/journal.pone.0027939)
Supplement: Table S1 — Studies of the effects of social stressors and/or social status on the development of CAD in non-human primates. (DOC) [file pone.0027939.s003.doc]

Table S1: Studies of the effects of social stressors and/or social status on the development of CAD in non-human primates

| **Study number*, author, year and purpose of study** | **Sample** | **Study design & intervention** | **Results relating to social status or CAD** |
| --- | --- | --- | --- |
| 1. Adams et al. (1985) [26]  To explore the influence of psychosocial factors on atherosclerosis in ovariectomised and intact monkeys | 52 preselected female cynomolgus monkeys fed atherogenic diet for 30 months (8 subsequently died of unrelated causes) | Controlled trial, ovariectomy (n=25) vs intact ovaries (n=27) | *Social status*: Ovariectomised dominant females had significantly more extensive CAA than intact females (0.25mm^2^ vs 0.04 mm^2^; p < 0.05). No significant association between dominance/ subordination and presence of large lesions, or between intact and Ovariectomised subordinates. Intimal area of coronary arteries:  1. Ovariectomised and dominant: 0.25mm^2^  2. Ovariectomised and subordinate: 0.15 mm^2^  3. intact and dominant: 0.04 mm^2^  4. intact and subordinate: 0.14 mm^2^ |
| 2. Clarkson et al. (1990) [5]  To examine effect of oral contraceptives on atherosclerosis | Female cynomolgus monkeys on moderately atherogenic diet (N=83; 10 subsequently died) | Controlled trial: 2 groups randomised to different oral contraceptives; one group randomised to control | *Social status*: Pre-experimental social status predicted atherosclerosis on necropsy (p<0.03, no other data), controlling for LDL and HDL; direction of relationship unclear |
| 3. Hamm (1983) [20,50]  To examine the effect of gender and social stress on coronary artery atherosclerosis | 16 male and 16 female feral adult Macaca fascicularis monkeys | Randomly allocated to social groups, then housed in stable groups of 4 animals of one sex for 16 months. Fed moderately atherosclerotic diet. | *Social status*: Coronary artery lumen stenosis : males had more CAA than females and submissive animals had more extensive CAA than dominant animals. Mean % stenosis (standard error of mean):  1. male dominant: 23.1% (8) (n=8)  2. male submissive: 44.4% (6.9) (n=8)  3. female dominant: 6.9% (3.4) (n=8)  4. female submissive: 24.0% (7.4) (n=8)  2x2 (gender by competitiveness) ANOVA:  Gender: F = 8.14; p = 0.008. Dominance: F = 10.12; p = 0.004. No gender x competitiveness interaction (p > 0.05). Positive results also for atherosclerosis in aorta and R iliac artery |
| 4. Kaplan et al. (1982) [27]  To examine the effects of social environment and social status on atherosclerosis | 30 Male cynomolgus monkeys | Controlled trial: monkeys assigned to unstable or stable social groups (i.e. 2 experimental conditions, 15 monkeys in each condition) | *Social status*: No significant main effect for dominance or stable/unstable. Dominant monkeys in unstable social condition had greater mean atherosclerosis than dominants in stable conditions (0.74mm^2^ vs 0.32mm^2^;  p=0.034); Severity of atherosclerosis was greater in unstable dominants than unstable subordinates (0.74mm^2^ vs 0.38mm^2^; p<0.05). |
| 5. Kaplan et al. (1983) [29,30] | 30 Male cynomolgus monkeys (different from above) | Controlled trial, two groups:  1. stressed: periodically re-organised groups (n=15)  2. unstressed (n=15)  Randomly allocated to three 5-member groups for 21 months. Fed a low fat, low cholesterol diet | *Stress:* Stressed animals had larger intimal areas (greater CAA) than controls (p < 0.002).  More animals in stressed group classified as grade 2 or more (greater CAD) on extent of change in coronary arteries than in controls (11/15 vs 4/14; p= 0.02). No sig. differences in total serum cholesterol, HDLC, BP, blood glucose. |
| 6. Kaplan et al, (1984) [21]  To investigate the role of psychosocial and reproductive factors in atherogenesis | 23 female and 15 male cynomolgus monkeys | Controlled trial: 2 all-male, stable groups, and 2 female groups, one stable, one deliberately socially disrupted every 12 weeks for 2 years. Females were housed in 4/5 member groups with a vasectomised male (who was not included in the study). Fed atherogenic diet for 30 months; 4 died of unrelated causes before the end of the study. | *Social status*: Significantly greater CAA in males compared to dominant females (p =0.021) and subordinate significantly more likely to have CAA (p = 0.007). No difference between males and subordinate females (p>0.10), or between stable and unstable groups (data not presented). |
| 7. Kaplan et al. (1987) [22]  To study the effect of propranolol on dietary induced coronary artery atherosclerosis | 30 male cynomolgus monkeys (24 contribute data) | Controlled trial: propranolol (n=15) vs untreated (n=15)  Randomised into 5 member social groups re-organised monthly (with oestrogen implanted female). fed moderately atherogenic diet for 26 months. | *Social status*: Coronary artery atherosclerosis (plaque area/mm ^2^)  Untreated dominant monkeys had significantly more CAA than other groups p < 0.05mm ^2^  1. propranolol treated dominant: 0.23mm ^2^  2. untreated dominant: 0.71mm ^2^  3. untreated subordinate: 0.3mm ^2^  4. propranolol treated subordinate: 0.43mm ^2^  ANOVA drug x dominance: no main effect but significant drug treatment x dominance interaction (F_1,20_ = 5.48, p = 0.028). |

| 8. Kaplan et al. (1993) [31]  To study the effects of dietary and social manipulation in male monkeys with established atherosclerosis | 83 adult male cynomolgus monkeys (started with 100; 17 died) | Controlled trial; groups balanced for plasma cholesterol response to diet. Baseline period with stable social groups; fed atherogenic diet for 14 months pre-entry.  Psychosocial stressor: unstable environment due to group reorganisation  1. Baseline (n=21 necropsied)  Others followed up for further 28 months  2. HFHC/STRESS (high-fat, high-cholesterol, unstable social environment) (n=18), vs  3. LFLC/STRESS (low fat, low cholesterol/ unstable social environment) (n=21), vs  3. LFLC/NO STRESS (low-fat, low-cholesterol/ stable social environment) (n=23) | *Social stress:* High fat, stressed monkeys had more CAA (larger lesions) than the other groups. No difference between other 3 groups  Coronary lesions (mm^2^, estimated from graph)  1. baseline: 0.4 mm^2^  2. HFHC/STRESS: 0.9 mm^2^  3. LFLC/STRESS: 0.35 mm^2^  4. LFLC/NO STRESS: 0.3 mm^2^  Post hoc contrasts showed HFHCC/Stress group lesions significantly larger |
| --- | --- | --- | --- |
| 9. Kaplan & Manuck (2001,2002) [22, 51,52]  To determine whether pre-menopausal social status predicts post-menopausal atherosclerosis | 175 female cynomolgus monkeys | Fed for 2 years on atherogenic diet, following baseline period. 26 month treatment period, in which half the social groups randomly received oral contraceptives. Monkeys then oophorectomised, then 36 post-menopausal period (again with atherogenic diet ), followed by necropsy and assessment of CAD. | *Social status*: Main effect for status (F1,165=8.82, p=0.01). In planned comparisons, untreated dominant monkeys had less CAD than untreated subordinates (p=0.001). This increase was inhibited by exogenous oestrogen (p<0.01). |
| 10. Shively et al. (1989, 1990) [22,53]  To compare plasma lipids and coronary artery atherosclerosis in monkeys housed in single-cages with those housed in social groups. | 77 wild-caught female cynomolgus monkeys | 2 x 2 controlled trial: Single cage vs social housing, and oral contraceptive (Ovral) vs no OC. Fed atherogenic diet for 7 months pre-treatment and 24 months (experimental period).  1. single cage untreated (n=15)  2. single cage, treated with OC (n=15)  3. social housing, untreated (n=24)  4. social housing, treated with OC (n=23) | *Social status*: Within the untreated controls, single caged monkeys had significantly more CAA than socially dominant females (p < 0.05) but not socially subordinate females (p > 0.10)  1. dominant in social groups: 0.01 mm^2^  2. subordinate in social groups: 0.05 mm^2^  3. housed in single cages: 0.124 mm^2^. Less carotid atherosclerosis in dominant females (p=0.04).  Females housed in single cages had significantly more CAA than those in social housing (F_1,73_ = 6.6, p = 0.01). Mean intimal area (estimated from graphs):  1. single cage untreated: 0.125 mm^2^  2. single cage, treated with OC: 0.05 mm^2^  3. social housing, untreated: 0.024 mm^2^  4. social housing, treated with OC: 0.018 mm^2^ |
| 11. Shively & Clarkson (1994) [28]  To examine the effect of manipulation of social environment on coronary artery atherosclerosis | 48 adult female cynomolgus monkeys (6 died for reasons unrelated to study). | Fed atherogenic diet, housed in social housing for 1 month, then single cages for 3 months pre-experiment.  Monkeys randomly allocated to 4-member groups for 8 wks until social status stabilised. Then dominant monkeys housed together and subordinates housed together forming 4 groups for 26 months:  1.Females that were initially dominant, and remained dominant after regrouping (n=11)  2. Initially dominant, subordinate after regrouping (n=11)  3. Initially subordinate, became dominant (n=8)  4. Initially subordinate, remained subordinate (n=12) | *Social status*: Significant interaction between initial social status and manipulated social status (p < 0.03). Among initially subordinate females, those becoming dominant had more extensive CAA and among initially dominant females, those who became subordinate had more extensive CAA  Mean plaque area/ mm ^2^  1.Initially dominant, and remained dominant after regrouping: 0.03mm ^2^  2. Initially dominant, became subordinate after regrouping: 0.19mm ^2^  3. Initially subordinate, became dominant: 0.09mm ^2^  4. Initially subordinate, remained subordinate: 0.04mm^2^ |
| 12. Williams et al. (1991)[32]  To examine the effect of chronic psychosocial disruption and diet on dilator responses of coronary arteries | 33 adult male cynomolgus monkeys | Experimental study: 4 groups:  1. High cholesterol diet, with social disruption (n=9);  2. Low cholesterol diet, with social disruption (n=8)  3. Low cholesterol diet, without social disruption (n=10)  4. Controls (n=6) | Data only presented for low cholesterol group (Table 1 in paper). Groups 2 vs 3, mean plaque size=0.5mm^2^ vs 0.4mm^2^ (t=0.45, p=0.65). |
| 13. Williams et al. (1994) [24]  To identify determinants of coronary artery reactivity among pre-menopausal female monkeys, in order to identify factors that may underlie angina in women | 25 adult female cynomolgus monkeys | Fed atherogenic diet. Held in single cages for 3 months pre-entry Randomly allocated to 4-member groups (duration not stated) in which social status stabilised. | *Social status*: Coronary artery atherosclerosis: No significant difference in mean intimal area between dominant and subordinate status (0.2 vs 0.25 mm ^2^ ; p > 0.05), or in TPC or HDL concentrations, or SBP or DBP |
| 14. Williams et al. (2003)[33]  To investigate effects of exercise on atherosclerosis | 71 male cynomolgus monkeys | 2x2 factorial design: exercise (running, sedentary) vs social instability (stable/unstable social groups, regularly reorganised). All fed high-fat diet to model North American diet | No significant main effect of social reorganisation on intimal area or lumen area (p<0.05) (data not presented; stable vs unstable 0.78mm^2^ vs 0.94 mm^2^, estimated from Fig. 2). |

* **These are the study numbers which are used in the Results section of the main text**

*Key:*

CAA: coronary artery atherosclerosis; CAD: coronary artery disease; SBP/DBP: Systolic/diastolic blood pressure; HDLC: High density lipoprotein cholesterol concentration; TPC: Total plasma cholesterol; propranolol: beta-blocking drug used to control e.g., angina, which reduces heart rate and blood pressure
