# Supplementary material for: The Monkey Puzzle: A Systematic Review of Studies of Stress, Social Hierarchies, and Heart Disease in Monkeys
Source: PLoS One. 2012 Mar 21;7(3):e27939. doi: 10.1371/journal.pone.0027939 (PMC3309950; doi:10.1371/journal.pone.0027939)
Supplement: Table S2 — Examples of quotations from papers citing primate studies. (DOC) [file pone.0027939.s004.doc]

| **Table S2: Examples of quotations from papers citing primate studies** |
| --- |
| **Examples of citations of primate studies linking the Shively and Sapolsky studies:**  Nordstrom et al. (2001)^37^: “This interpretation is supported by the finding in primates that stress promotes atherosclerosis in both males and females.*^(i,ii)^*  *References*: (*i).Kaplan (1982); (ii).Shively & Clarkson (1994)*  Wilkinson (1997)^7^: “Animal models have shown, subordinate social status has health consequences even when the physical environment and diet are invariant” *^(i), (ii)^*  *References: (i). Shively & Clarkson (1994); (ii). Sapolsky (1993)*  **Examples of “five-fold” claims for effect of downward mobility in social hierarchy on atherosclerosis:**  “The effects of manipulating social status were dramatic. Dominants who became subordinate had a fivefold excess of coronary plaques compared with animals who remained dominant.” ^54^  “Loss of social status resulting from being rehoused with more dominant animals was associated with fivefold increases in coronary artery atherosclerosis.” ^7^  “Downwardly mobile animals showed a fivefold increase in atherosclerosis over two years*.*”^7^ |

**Additional reference**

54. Brunner E. (1997) Socioeconomic determinants of health: stress and the biology of inequality. Br Med J 314:1472.
